# Influence of physical activity in asthmatic children

**DOI:** 10.1186/2045-7022-3-S1-P4

**Published:** 2013-05-03

**Authors:** Giancarlo Tancredi, Ilaria Ernesti, Annalisa di Coste, Laura Schiavi, Flavia Tubili, Flaminia Bardanzellu, Valentina De Vittori, Marzia Duse

**Affiliations:** 1 Sapienza Università di Roma, Policlinico Umberto I, Italy; 2 Sapienza Università di Roma, Policlinico Umberto I, Dipartimento di Immuno-Allergologia Pediatrica, Italy

## Aim of the study

This preliminary study investigated the influence of physical activity levels on lung function tests, exercise test and differences of preparticipation screening (noncompetitive or competitive) in asthmatic children and controls.

## Methods

We compared functional respiratory testing in 72 asthmatic children (mean age 11.4±2.6 years) and in 70 healthy subjects (mean age 12.5±2.5 years). Each group was divided in 2 subgroups using a physical activity level cut-off (< 2 or > 3 hours spent for week), that way generating 4 study groups: asthmatic trained children, non asthmatic trained children, trained controls, non trained controls. We investigated type of preparticipation screening (noncompetitive or competitive) and functional characterizes. All subjects underwent a maximal treadmill exercise test, determining maximal oxygen uptake by indirect method, metabolic equivalents and exercise time, and performed spirometry pre and post exercise.

## Results

No significant differences were found on spirometry between asthmatic children and controls. Exercise testing showed, respectively for controls and asthmatic subjects, metabolic equivalents 15.2±2.7; 13.0±2.7 (p<0.0001); maximal oxygen uptake 52.2±9.9; 44.6±8.7 ml/kg/min (p<0.0001); exercise time 12.1±2.2; 10.5±2.4 min (p<0.0001). Competitive screening preparticipation was performed in 45.7% of controls and 22.2% of asthmatic subjects, noncompetitive screening in 54.3% of controls and 77.8% of asthmatic children (p<0.003).

## Conclusions

Physical activity influences exercise parameters (exercise time, METS, VO2max) in asthmatic children: asthmatic trained children, with asthma controlled, have functional parameters better than non trained controls and similar to trained controls.

